# Genetic Cell-Surface Modification for Optimized Foam Fractionation

**DOI:** 10.3389/fbioe.2020.572892

**Published:** 2020-10-29

**Authors:** Christian C. Blesken, Isabel Bator, Christian Eberlein, Hermann J. Heipieper, Till Tiso, Lars M. Blank

**Affiliations:** ^1^iAMB - Institute of Applied Microbiology, ABBt - Aachen Biology and Biotechnology, RWTH, Aachen University, Aachen, Germany; ^2^Bioeconomy Science Center (BioSC), Forschungszentrum Jülich GmbH, Jülich, Germany; ^3^Department of Environmental Biotechnology, UFZ - Helmholtz Centre for Environmental Research, Leipzig, Germany

**Keywords:** rhamnolipid, 3-(3-hydroxyalkanoyloxy)alkanoic acid (HAA), integrated product recovery, foam fractionation, cell surface hydrophobicity, large adhesion protein, flagellum, metabolic engineering

## Abstract

Rhamnolipids are among the glycolipids that have been investigated intensively in the last decades, mostly produced by the facultative pathogen *Pseudomonas aeruginosa* using plant oils as carbon source and antifoam agent. Simplification of downstream processing is envisaged using hydrophilic carbon sources, such as glucose, employing recombinant non-pathogenic *Pseudomonas putida* KT2440 for rhamnolipid or 3-(3-hydroxyalkanoyloxy)alkanoic acid (HAA, i.e., rhamnolipid precursors) production. However, during scale-up of the cultivation from shake flask to bioreactor, excessive foam formation hinders the use of standard fermentation protocols. In this study, the foam was guided from the reactor to a foam fractionation column to separate biosurfactants from medium and bacterial cells. Applying this integrated unit operation, the space-time yield (STY) for rhamnolipid synthesis could be increased by a factor of 2.8 (*STY* = 0.17 g_RL_/L·h) compared to the production in shake flasks. The accumulation of bacteria at the gas-liquid interface of the foam resulted in removal of whole-cell biocatalyst from the reactor with the strong consequence of reduced rhamnolipid production. To diminish the accumulation of bacteria at the gas-liquid interface, we deleted genes encoding cell-surface structures, focusing on hydrophobic proteins present on *P. putida* KT2440. Strains lacking, e.g., the flagellum, fimbriae, exopolysaccharides, and specific surface proteins, were tested for cell surface hydrophobicity and foam adsorption. Without flagellum or the large adhesion protein F (LapF), foam enrichment of these modified *P. putida* KT2440 was reduced by 23 and 51%, respectively. In a bioreactor cultivation of the non-motile strain with integrated rhamnolipid production genes, biomass enrichment in the foam was reduced by 46% compared to the reference strain. The intensification of rhamnolipid production from hydrophilic carbon sources presented here is an example for integrated strain and process engineering. This approach will become routine in the development of whole-cell catalysts for the envisaged bioeconomy. The results are discussed in the context of the importance of interacting strain and process engineering early in the development of bioprocesses.

## Introduction

Bio-based materials such as biosurfactants are in high demand (Müller et al., 2012), as their use potentially lowers the carbon footprint compared to fossil-based surfactants. Biosurfactants like rhamnolipids and derivatives can be utilized for a wide range of industrial applications, e.g., in the chemical, cosmetic, pharmaceutical, and food industries, as well as for bioremediation of polluted soils and enhanced oil recovery (Banat et al., 2000; Singh et al., 2007; Kosaric and Vardar-Sukan, 2015). Latest publications discuss the use of hydroxyalkanoyloxy alkanoates (HAAs), representing the hydrophobic moiety of rhamnolipids (RLs), for the conversion to biofuel (Meyers et al., 2019), secondary alcohols and linear alkanes (Mensah et al., 2020), or polyurethane (Tiso et al., 2020b).

We previously designed and constructed recombinant *Pseudomonas putida* KT2440 strains able to synthesize mono-rhamnolipids and HAAs (Wittgens et al., 2011). HAA, a dimer of ACP-activated β-hydroxydecanoates, is synthesized by the enzyme 3-hydroxyacyl-ACP:3-hydroxyacyl-ACP O-3-hydroxy-acyl-transferase (RhlA). RhlA determines to a large extend the carbon chain lengths of HAA molecules (Cabrera-Valladares et al., 2006; Germer et al., 2020). Rhamnolipids are synthesized by the rhamnosyltransferase I (RhlB), forming a glycosidic bond between the HAA, and a rhamnose. The hydrophobic hydroxyl fatty acid dimer and the hydrophilic sugar account for the amphiphilic nature of the rhamnolipids. However, not only rhamnolipids, but also the aglyconic HAAs feature tensioactive properties (Deziel et al., 2003). While vegetable oils are favored for rhamnolipid production with the native host *P. aeruginosa* (Müller and Hausmann, 2011), the recombinant production strains allow rhamnolipid production from sugars like glucose or xylose (Wittgens et al., 2011; Bator et al., 2020).

Microbial growth and the production of rhamnolipids depend on the availability of nutrients and oxygen in the culture broth. In shake flask cultures, surfactant synthesis requires minimal technical effort, but the oxygen transfer into the culture is limited (Meier et al., 2016). For comprehensive process control, sufficient oxygen transfer, and enhanced scalability, the use of bioreactors is essential. Under these conditions, rhamnolipids and HAAs adsorb to the surface of gas bubbles with their hydrophobic moiety. This adsorption is an energy-dependent process, reducing the surface tension of the gas bubble and therefore decreases the Gibbs free energy of the system (Stevenson and Li, 2014). Thereby, the bubbles are stabilized, resulting in foaming. Consequently, a foam highly enriched with surfactants builds up in the headspace of the bioreactor. An associated accumulation of bacterial cells and medium in the foam causes a loss of homogeneity in the reactor. As homogeneous conditions in the reactor assure an optimal mass and energy transfer, process efficiency declines (Etoc et al., 2006; Lara et al., 2006). Furthermore, foam formation can result in obstruction of filters, valves, tubing, as well as sterility problems in the fermenter (Delvigne and Lecomte, 2010). To avoid foam formation, the application of detergents with antifoam activity helps to stabilize microbial rhamnolipid production (Beuker et al., 2016a). However, adding large amounts of antifoam increases production costs and hampers downstream processing (DSP) (Ochsner et al., 1996).

Approaches to reduce foam formation consider specific gassing membranes, a lowered pH value, or the introduction of an organic phase (Chayabutra and Ju, 2001; Kronemberger et al., 2008, 2010; Sodagari and Ju, 2013). As DSP accounts for the majority of the process costs, efficient product purification approaches need to be considered (Heyd, 2008; Najmi et al., 2018). DSP and foaming have been identified as the main challenges for cost-competitive production of biosurfactants (Mukherjee et al., 2006; Winterburn et al., 2011; Winterburn and Martin, 2012). However, foaming can also be an *in situ* product removal technique. In a foam fractionation column, the denser culture medium drains through the foam back into the reactor. It is only retarded by the shear force experienced at the gas-liquid interface (Stevenson and Li, 2014). For microbial rhamnolipid production, foam fractionation is reported (Siemann and Wagner, 1993; Heyd et al., 2011; Beuker et al., 2016b; Anic et al., 2018). Foam fractionation is a cost-effective technology that requires only simple technical installations. However, for efficient *ex* or *in situ* foam fractionation, the loss of biocatalyst from the reactor due to biomass accumulation in the foam is a challenge. Foam adhesion of cells might be influenced by the cell surface hydrophobicity (CSH).

So far, the CSH of *Pseudomonas* strains is mainly discussed in the context of biofilm formation and general stress adaptation (Heipieper et al., 2007; Baumgarten et al., 2012a; Eberlein et al., 2018). In *Pseudomonas*, several cell surface molecules contributing to changes in CSH have been identified, such as the lipopolysaccharide layer (LPS) (Makin and Beveridge, 1996; Kobayashi et al., 2000). *Pseudomonas putida*'s large adhesive protein A (Lap A) and particularly F (LapF) increase cell surface hydrophobicity (Lahesaare et al., 2016). In *P. putida* KT2440, LapA is the largest surface protein and required for cell-to-cell as well as for abiotic surface interactions (Hinsa et al., 2003; Fuqua, 2010). LapF is the second largest surface protein with a key role in the development of a mature biofilm (Martinez-Gil et al., 2010). Additionally, it was shown that the release of outer membrane vesicles (OMV) as a general stress response mechanism increases CSH in *P. putida* (Baumgarten et al., 2012b). For the non-flagellated *P. putida* KT2440, a significantly lowered surface hydrophobicity was determined (Martinez-Garcia et al., 2014).

We present rhamnolipid and HAA production with recombinant *P. putida* KT2440 in a bioreactor equipped with a foam fractionation column. The challenge of cell loss during foam discharge was tackled by strain engineering. More than ten different cell surface structure deletion mutants were tested for lowered CSH. In a newly established experimental setup, the correlation of a reduced CSH with a lower tendency for cell enrichment in the foam was confirmed. Here, especially the deletion of the flagellar machinery, LapF, and LapF in combination with LapA reduced biomass adhesion to the foam. Indeed, cell surface engineered strains allowed stable rhamnolipid and HAA production using foam fractionation for integrated product removal from the bioreactor.

## Materials and Methods

### Bacterial Strains and Plasmids

All bacterial strains and plasmids used in this study are listed in Table 1. The deletion mutants were constructed by using the I-SceI-system described by Martinez-Garcia and de Lorenzo (2011). Briefly, 500–800 bp upstream and downstream flanking regions of the target sites were amplified from the genomic DNA of *P. putida* KT2440 and cloned into the non-replicative pEMG (Km^R^) or pSEVA512S (Tc^R^) vector. The resulting plasmids were transferred into *Escherichia coli* PIR2 and conjugated into *Pseudomonas* strains via triparental mating. The plasmid pSW-2, encoding for the I-SceI restriction enzyme, was transformed to allow for the deletion of the gene locus of interest. Positive colonies sensitive for kanamycin or tetracycline were verified for targeted deletion by colony polymerase chain reaction (PCR). To obtain marker-free clones, the recombinant strains were cured of pSW-2 plasmid by re-inoculation in lysogeny broth (LB) medium without gentamycin and verified again by colony PCR (Supplementary Figure 1). After verification via colony PCR, the single deletion strains were sequenced by Eurofins Genomics (Ebersberg, Germany) to exclude mutations. In this study, twelve knock-out mutants were engineered (Table 1).

**Table 1:** Bacterial strains and plasmids used in this study.

**Strains and plasmids**	**Characteristics**	**References or sources**
***E. coli***		
PIR2	F^−^, Δ*lac169, rpoS*(*Am*), *robA1, creC510, hsdR514, endA, recA1 uidA*(Δ*MluI*)::pir; host for *oriV*(R6K) vectors	ThermoFisher Scientific
PIR2 pBG14ffg	PIR2 harboring Tn7 delivery vector pBG14ffg; containing BCD2-*msfgfp* fusion	Köbbing et al., 2020
HB101 pRK2013	Sm^R^, *hsdR-M^+^, proA2, leuB6, thi-1, recA*; harboring plasmid pRK2013	Ditta et al., 1980
PIR2 pKS03	PIR2 harboring Tn7 delivery vector pKS03 for chromosomal integration; containing *rhlA* from *P. aeruginosa* PA01; pBG derivative	This study
PIR2 pEMG-pvd	PIR2 harboring plasmid pEMG-pvd	This study
PIR2 pEMG-flag1	PIR2 harboring plasmid pEMG-flag1	This study
PIR2 pEMG-flag2	PIR2 harboring plasmid pEMG-flag2	This study
PIR2 pEMG-alg	PIR2 harboring plasmid pEMG-alg	This study
PIR2 pEMG-bcs	PIR2 harboring plasmid pEMG-bcs	This study
PIR2 pEMG-pea	PIR2 harboring plasmid pEMG-pea	This study
PIR2 pSEVA512S-peb	PIR2 harboring plasmid pSEVA512S-peb	This study
PIR2 pEMG-*lapA*	PIR2 harboring plasmid pEMG-*lapA*	This study
PIR2 pEMG-*lapF*	PIR2 harboring plasmid pEMG-*lapF*	This study
DH5αλpir pΔpha	DH5αλpir harboring plasmid pΔpha	Mato Aguirre, 2019
DH5α pSW-2	DH5α harboring plasmid pSW-2 encoding I-SceI nuclease, tool for genomic deletion	Martinez-Garcia and de Lorenzo, 2011
DH5αλpir pTNS1	DH5αλpir harboring plasmid pTNS1	Choi et al., 2005
DH5αλpir pSK02	DH5 αλpir harboring Tn7 delivery vector pSK02 for chromosomal integration; containing *rhlAB* genes from *P. aeruginosa* PA01	Bator et al., 2020
***P. taiwanensis***		
VLB120	wild type	Panke et al., 1998
***P. putida***		
DOT-T1E	wild type	Ramos et al., 1998
S12	wild type	Hartmans et al., 1990
KT2440	wild type	Bagdasarian et al., 1981
KT2440 Δflag	Δ*PP_4328*-PP_*4344*, ΔPP_*4351*-PP_*4397* deletion of flagellum operon	This study
KT2440 Δalg	ΔPP_*1277*-PP_*1288* deletion of alginate operon	This study
KT2440 Δbcs	ΔPP_*2634*-PP_*2638* deletion of cellulose operon	This study
KT2440 Δpea	ΔPP_*3132*-PP_*3142* deletion of exopolysaccharide a operon	This study
KT2440 Δpeb	ΔPP_*1795*-PP_*1788* deletion of exopolysaccharide b operon	This study
KT2440 Δ*lapA*	ΔPP_*0168* deletion of large adhesion protein A operon	This study
KT2440 Δ*lapF*	ΔPP_*0806* deletion of large adhesion protein F operon	This study
KT2440 Δ*lapA*Δ*lapF*	ΔPP_*0168*, ΔPP_*0806* cumulative deletion of *lapA* and *lapF*	This study
KT2440 Δpha	ΔPP_*5003*-PP_*5008* deletion of polyhydroxyalkanoate operon	This study
KT2440 ΔfimbriaeΔpili	ΔPP_*1887*-PP_*1891*, ΔPP_*2357*-PP_*2363*, ΔPP_*4986*-PP_*4992*, ΔPP_*5080*-PP_*5083*, ΔPP_*0607*-PP_*0611* deletion of fimbriae and pili operon	BacMine S. L., unpublished
KT2440 ΔfimbriaeΔpiliΔcurli	ΔPP_*1887*-PP_*1891*, ΔPP_*2357*-PP_*2363*, ΔPP_*4986-*PP_*4992*, ΔPP_*5080*-PP_*5083*, ΔPP_*0607*-PP_*0611*, ΔPP_*3472*-PP_*3484*, ΔPP_*1993*, ΔPP_*5093* deletion of fimbriae, pili and curli operon	BacMine S. L., unpublished
KT2440 GR20	ΔPP_*4219*-PP_*4221*, ΔPP_*4328*-PP_*4344*, ΔPP_*4351*-PP_*4397*, ΔPP_*1277*-PP_*1288*, ΔPP_*2634*-PP_*2638*, ΔPP_*3132*-PP_*3142*, ΔPP_*1795*-PP_*1788*, ΔPP_*0168*, ΔPP_*0806*, ΔPP_*5003*-PP_*5008* cumulative deletion of *lapA* and *lapF*, pyoverdine, flagellum, alginate, cellulose, exopolysaccharide a & b, polyhydroxyalkanoate operon	This study
KT2440 KS3	att*Tn7*::P_ffg_-*rhlA*	This study
KT2440 SK4	att*Tn7*::P_ffg_-*rhlAB*	Tiso et al., 2020a
KT2440 Δ*lapF*_HAA	*P. putida* KT2440 Δ*lapF* with att*Tn7*::P_ffg_-*rhlA*	This study
KT2440 Δ*lapF*_RL	*P. putida* KT2440 Δ*lapF* with *att*Tn7::P_ffg_-*rhlAB*	This study
KT2440 Δ*lapA*Δ*lapF*_RL	*P. putida* KT2440 Δ*lapA*Δ*lapF* with *att*Tn7::P_ffg_-*rhlAB*	This study
KT2440 Δflag_RL	*P. putida* KT2440 Δflag with *att*Tn7::P_ffg_-*rhlA*	This study
KT2440 GR20_RL	KT2440 GR20 with *att*Tn7::P_ffg_-*rhlAB*	This study

The construction of the rhamnolipid production strains was performed by using the mini-Tn7 delivery transposon vector pSK02 as described previously (Bator et al., 2020). Mono-rhamnolipid producing clones were identified using cetrimide-blood agar plates (7.5% (v/v) sheep blood, Fiebig-Nährstofftechnik, Idstein-Niederauroff, Germany).

The HAA production strain was constructed using vector pKS03, which was generated based on pSK02. Using primers KS08 and KS02 a DNA fragment (4,393 bp) was obtained consisting of all the elements of pSK02 without the *rhlB* gene but still containing the gene enabling HAA production (*rhlA*) from *P. aeruginosa*. This DNA fragment was circularized using Gibson Assembly. The resulting mini-Tn7 vector pKS03 was transferred by mating and integrated into the *att*Tn7 site, according to Zobel et al. (2015). For mating, the recipient strain (*Pseudomonas*), helper strain *E. coli* HB101 pRK2013, transposase-leading strain *E. coli* DH5αλpir pTNS1, and donor strain *E. coli* DH5α PIR2 pKS03 were used. Mating procedures were performed according to a streamlined method (Wynands et al., 2018). All used primers for the construction of plasmids and verification of deletion mutants are listed in Supplementary Table 1.

### Culture Conditions

All strains were stored at −80°C in a 20% (v/v) glycerol solution as cryo-culture. For cultivation, frozen cells were transferred to LB agar (10 g/L tryptone, 5 g/L yeast extract, 10 g/L NaCl, 20 g/L agar). If required, 50 mg/L ampicillin, 25 mg/L gentamicin, or 50 mg/L kanamycin was added to the medium for selection and to prevent contamination. After mating procedures, *P. putida* strains were selected on cetrimide agar (Sigma-Aldrich Corp., St. Louis, MO, USA). Cells from LB agar were transferred to 5 mL LB medium in a test tube and shaken at 200 rpm with a 50 mm shaking diameter at 30°C. After 15 h, 50 mL minimal medium with the corresponding antibiotic and glucose concentration were inoculated at a specific optical density at 600 nm (OD_600_), corresponding to the respective experiment. The 500 mL flasks were shaken at 300 rpm with a 50 mm shaking diameter at 30°C (Multitron Pro, Infors AG, Bottmingen, Switzerland). As minimal medium, the mineral salt medium (MSM) according to Hartmans et al. (1989) was applied with a modified phosphate buffer at pH 7. For shake flask cultivation, 11.64 g K_2_HPO_4_ and 4.89 g NaH_2_PO_4_ were used (per L). In fermenters with pH control via 30% (v/v) NH_4_OH, 3.88 g K_2_HPO_4_, and 1.63 g NaH_2_PO_4_ were applied per L. Further medium components were (per L) 2 g (NH_4_)_2_SO_4_ and the trace elements 10 mg EDTA, 0.1 mg MgCl_2_·6 H_2_O, 2 mg ZnSO_4_·7 H_2_O, 1 mg CaCl_2_·2 H_2_O, 5 mg FeSO_4_·7 H_2_O, 0.2 mg Na_2_MoO_4_·2 H_2_O, 0.2 mg CuSO_4_·5 H_2_O, 0.4 mg CoCl_2_·6 H_2_O, and 1 mg MnCl_2_·2 H_2_O.

### Bacterial Foam Adhesion

**Figure 1:**
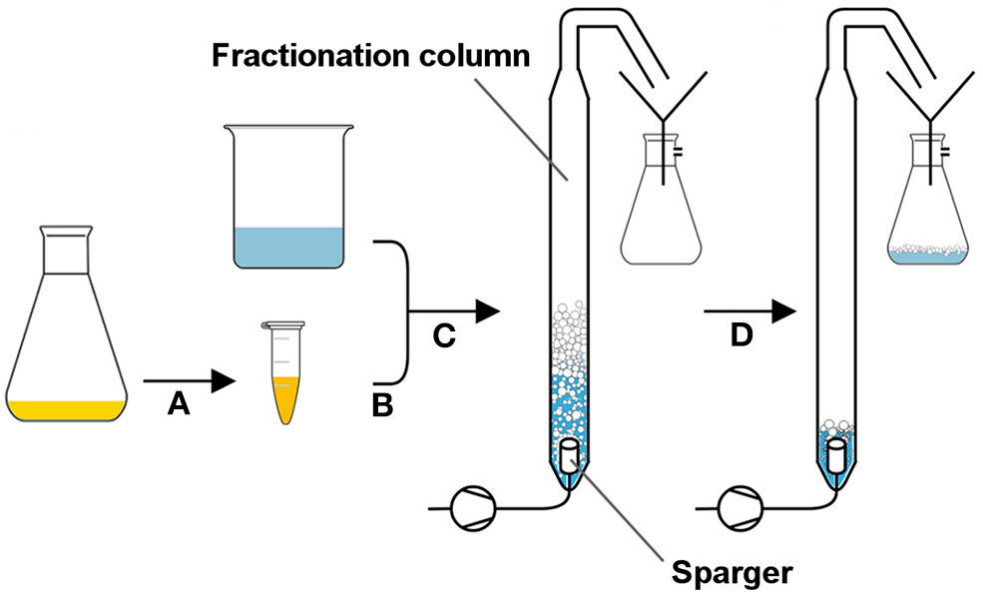
Foam fractionation setup to determine cell agglomeration in foam. The culture broth was centrifuged, and the pellet was washed **(A)** before it was resuspended in an isotonic solution containing rhamnolipids **(B)**. The cell suspension was filled into the fractionation column, and the gas flow was turned on **(C)**. The foam leaving the upper opening was collapsed by transfer through a thin tube and collected as foamate in a vacuum bottle **(D)**.

In order to determine biomass flotation characteristics in foam, cells were cultivated in shake flasks (start OD_600_ = 0.01, MSM, 10 g/L glucose) and harvested in the late exponential phase, at optical densities in between OD_600_ 4 and 6. A defined amount of biomass was washed twice with 25 mL 0.9% (w/v) NaCl. The pellet was resuspended in 37 mL 0.9% (w/v) NaCl to reach an OD_600_ of about 2. Subsequently, 3 mL of a purified aqueous rhamnolipid solution was added to the suspension to reach a final rhamnolipid concentration of 0.6 g_RL_/L. Before the fractionation, the pH values of all suspensions ranged from 6.2 to 6.6. The used foam fractionation glass column (Ø_inner_ = 32 mm, h = 600 mm) was fixed in an upright position. The sparger mounted on the column bottom had a pore size of 20 μm (bbi-biotech GmbH, Berlin, Germany). As soon as the cell suspension was filled into the column, the air flow ${\overset{∙}{V}}_{g}$ was set to 5 L/h (corresponding to a gas superficial velocity *j*_*g*_= 10.36 cm/min) by a rotameter (RGC2422, Yokogawa GmbH, Ratingen, Germany) at an overpressure of 0.5 bar. The foam raised to the upper column opening where it dropped into a funnel connected to a flask with a thin tube (Ø_inner_= 1 mm) to collapse the foam. The collection flask was set under low pressure (0.5 bar) using a vacuum pump (Type 115053, ILMVAC GmbH, Ilmenau, Germany). The fractionation was terminated after 30 min by turning off the gas flow. When the foam in the collection vessel (foamate) collapsed completely, samples were taken for OD_600_ measurement. The whole procedure is illustrated in Figure 1.

### Contact Angle Measurement

The evaluation of the surface hydrophobicity was carried out by the water contact angle measurement technique, as developed by Neumann et al. (2006). The strains were cultured in shake flasks (start OD_600_ = 0.1, MSM, 10 g/L glucose) until an OD_600_ > 0.5 was reached. The cells were harvested by centrifugation and resuspended in 1.8 mL 0.9% (w/v) NaCl. This washing step was repeated twice before 500 μL of the suspension was added to 19.5 mL 0.9% (w/v) NaCl solution. The suspension was vacuum filtered to obtain a cell lawn on the membrane filter (Ø_pores_ = 0.45 μm, Labsolute Th. Geyer GmbH & Co. KG, Renningen, Germany). The filter with the bacterial lawn was dried for 2 h at room temperature before the contact angle measurement was performed via the automated analysis system DSA100 with a DSA4 software package (A. Krüss Optronic GmbH, Hamburg, Germany). A 3 μL water droplet was placed on the bacterial lawn and the angle was measured after 80 ms.

### Fermentation Setup

**Figure 2:**
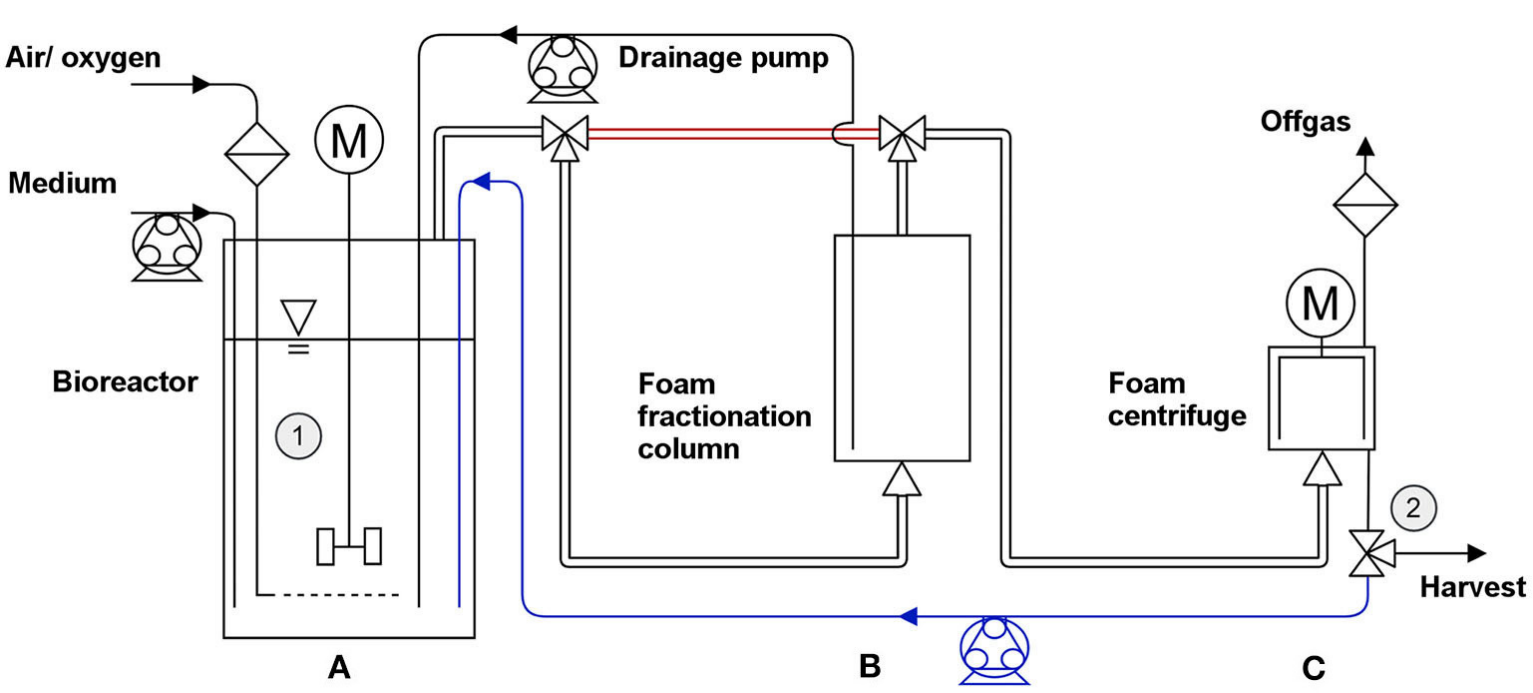
Fermentation setup with three stages in the growth phase and the harvest phase. Growth phase: 1st stage: no gassing into the stirred bioreactor **(A)**. 2nd stage: activated aeration; discharging foam through the exhaust directly into the foam centrifuge **(C)** (bypass in red); foamate reflux into reactor (blue). 3rd stage: bypass cut-off and integration of a foam fractionation column **(B)** between reactor and centrifuge. From the column, a pump returns the drained liquid while the fractionated foam is guided through an upper outlet. Harvest phase: product harvest by stopping the foamate reflux and collecting the foamate in a bottle. Regular refill of fresh medium to control working volume in the reactor. Sampling points are marked as ➀ (reactor) and ➁ (foamate outlet).

The fermentation was performed using a BioFlo 120 bioreactor system with a DASware control (Version 5.0) software package (both Eppendorf AG, Hamburg, Germany). The applied vessel with a total volume of 3 L was filled with 2 L minimal medium containing 20 g/L glucose. The conducted fermentation procedure was separated into two phases: 1. the growth phase to reach a defined biomass concentration and 2. the harvest phase. The stirred reactor (800 rpm) was inoculated with a preculture to an OD_600_ of 0.2. The here applied preculture was previously incubated for 12 h (start OD_600_ = 0.1, MSM, 20 g/L glucose) to gain an OD_600_ > 6. When the bioreactor culture reached an OD_600_ > 0.5, the gassing rate through a ring sparger was turned on (0.25 vvm) to prevent oxygen limitation. The dissolved oxygen (DO) was maintained at 30% by the addition of pure oxygen. The appearing foam left the reactor through the air exhaust into a foam centrifuge (Foamex 5 LS, Heinrich Frings GmbH & Co. KG, Rheinbach, Germany) collapsing the foam at 4,000 rpm. The foamate was pumped back into the reactor with 235 mL/min. After the foam formation exceeded the foamate reflux, the fractionation column (Ø_inner_ = 135 mm, h = 190 mm) with a drainage pump (${\overset{∙}{V}}_{\text{drainage }}=$ 50 mL/min) was introduced between the reactor air exhaust and the foam centrifuge. The stirring speed in the fermenter was reduced from 800 to 500 rpm. After 10 h of cultivation, 40 g glucose and trace elements (20 mg EDTA, 0.2 mg MgCl_2_·6 H_2_O, 4 mg ZnSO_4_·7 H_2_O, 2 mg CaCl_2_·2 H_2_O, 10 mg FeSO_4_·7 H_2_O, 0.4 mg Na_2_MoO_4_·2 H_2_O, 0.4 mg CuSO_4_·5 H_2_O, 0.8 mg CoCl_2_·6 H_2_O, and 2 mg MnCl_2_·2 H_2_O) were added to the broth. A second glucose feed was applied throughout the harvest period to prevent limitations. When a biomass concentration in the reactor of 5 g_CDW_/L was reached, the process was moved from the growth phase to the harvest phase. The surfactant harvest was initiated by stopping the foamate reflux into the reactor (Figure 2). Instead, the foamate leaving the system was collected and weighed. A gas superficial velocity of *j*_*g*_= 3.49 cm/min was reached in the fractionation column. The filling volume in the reactor was maintained at 2 L every 2 h by addition of fresh medium.

### Sampling and Processing

From shake flask cultivations, less than 750 μL sample were taken per sampling. From bioreactor cultivations, samples were taken from the reactor broth and the foamate. The OD_600_ was measured using an Ultrospec 10 cell density meter (Biochrom, Cambridge, UK). An OD_600_ of 1.0 corresponds with a determined cell dry weight, as listed in Supplementary Table 2. Glucose was analyzed as described previously (Hosseinpour Tehrani et al., 2019) in a Dionex Ultimate 3000 HPLC system, composed of the pump ISO-3100, the autosampler WPS-3000, and the column oven TCC-3000, connected to a DIONEX UltiMate 3000 Variable Wavelength Detector set to 210 nm (Thermo Fisher Scientific Inc., Waltham, MA, USA) and an RI detector SHODEX RI-101 (Showa Denko Europe GmbH, Munich, Germany) equipped with an ISERA Metab AAC 300 x 7.8 mm column (particle size: 10 μm, ISERA GmbH, Düren, Germany). The ammonium concentration in the culture supernatant was measured by a colorimetric method according to Willis et al. (1996), using salicylate and nitroprusside. For the determination of rhamnolipid and HAA concentrations, analytical methods and sample preparations were performed according to Bator et al. (2020), based on a method developed previously (Behrens et al., 2016; Tiso et al., 2016). Briefly, a RP-HPLC Ultimate 3000 HPLC system, composed of the pump LPG-3400, the autosampler WPS-3000, and the column oven TCC-3000, connected to a Corona Veo charged aerosol detector (CAD) (all Thermo Fisher Scientific Inc., Waltham, MA, USA) equipped with a NUCLEODUR C18 Gravity 150 × 4.6 mm column (particle size: 3 μm, Macherey-Nagel GmbH & Co. KG, Düren, Germany) was used. All components were identified via the retention time and quantified via the peak area compared to corresponding standards.

### Data Analysis

To define process parameters, the following equations were used: *X* is biomass, *S* is glucose, *P* is product, *t* is time, $\overset{∙}{V}$ is volume flow, *V* is volume, *A* is area, and *m* is mass. The yields of biomass from glucose (*Y*_*X*/*S*_) from ammonium ($Y_{X/{\text{NH}}_{4}^{+}}$), and the product yields from biomass (*Y*_*P*/*X*_) were determined for the shake flask cultivations for every defined time *t*_*i*_ by taking starting conditions at *t*_0_ as reference. The yields of product from glucose *Y*_*P*/*S*_ were always calculated for the total cultivation time. For the bioreactor applications, *m*_*P*_ is the sum of the mass of product in the reactor and in the separated foamate. *m*_*S*_ is defined as the mass of glucose remaining in the reactor and *m*_*S*_, _feed_ the glucose added during cultivation.

**Equation 1**

$$\begin{array}{l} \;Y_{X/S}\;(t_{i})\;=\;\frac{m_{X}\left(t_{i}\right)\;-\;m_{X}\left(t_{0}\right)}{m_{S}\left(t_{0}\right)}\;[{\text{g}}_{X}/{\text{g}}_{S}]\\ \;Y_{X/{\text{NH}}_{4}^{+}}\;(t_{i})\;=\;\frac{m_{X}\left(t_{i}\right)\;-\;m_{X}(t_{0})}{\;m_{{\text{NH}}_{4}^{+}}\left(t_{0}\right)\;-\;m_{{\text{NH}}_{4}^{+}}\left(t_{i}\right)}\;[{\text{g}}_{X}/{\text{g}}_{{\text{NH}}_{4}^{+}}]\\ \;Y_{P/X}\;(t_{i})\;=\;\frac{m_{P}\left(t_{i}\right)\;-\;m_{P}(t_{0})}{m_{X}\left(t_{i}\right)\;-\;m_{X}\left(t_{0}\right)}\;[{\text{g}}_{P}/{\text{g}}_{X}]\\ Y_{P/S,\text{ shake flask}}\;=\;\frac{m_{P}\;-\;m_{P}(t_{0})}{m_{S}\left(t_{0}\right)\;-\;m_{S\;}}\;[{\text{g}}_{P}/{\text{g}}_{S}]\\ \;Y_{P/S,\text{ reactor}}\;=\frac{m_{P}\;-\;m_{P}(t_{0})}{m_{S}\left(t_{0}\right)\;+\;m_{S,\text{ feed}}\;-\;m_{S\;}}\;[{\text{g}}_{P}/{\text{g}}_{S}] \end{array}$$

To characterize the STY, the formed product was divided by the corresponding volumes and cultivation times. For bioreactor experiments, the product separated with the foamate was taken into account.

**Equation 2**

$$\begin{array}{l} STY_{\text{shake flask}}=\;\frac{m_{P}\;-\;m_{P}\left(t_{0}\right)}{V_{\text{culture}}\cdot \;t}\;\left[{\text{g}}_{P}/\text{L}\cdot \text{h}\right]\\ \;STY_{\text{reactor}}=\;\frac{m_{P,\text{ reactor }}-\;m_{P,\text{ reactor}}\left(t_{0}\right)\;+\;m_{P,\text{ foamate}}}{V_{\text{culture}}\cdot \;t}\;\left[{\text{g}}_{P}/\text{L}\cdot \text{h}\right] \end{array}$$

For foam fractionation, the gas superficial velocity *j*_*g*_ is defined by Stevenson and Li (2014), with *A*_column_ as the sectional area of the specific column.

**Equation 3**

$$\begin{array}{l} j_{g}=\;\frac{{\overset{∙}{V}}_{g}}{A_{\text{column}}}\;\left[\text{m}/\text{s}\right] \end{array}$$

The biomass enrichment factors *E* are defined individually for the stand-alone bacterial foam adhesion experiments and the bioreactor experiments with foam fractionation. For the stand-alone experiments, the optical densities were multiplied with the correspondent foamate and initial volume to account for evaporation losses. For bioreactor experiments, enrichment factors were defined for each sampling point.

**Equation 4**

$$\begin{array}{l} \;E_{OD_{600}\cdot V}\;=\;\frac{OD_{\text{foamate}}\cdot V_{\text{foamate}}}{OD_{\text{initial}}\cdot V_{\text{initial}}}\;\left[-\right]\\ \;E_{\text{biomass}}\left(t_{i}\right)\;=\;\frac{OD_{\text{foamate}}\left(t_{i}\right)}{OD_{\text{reactor}}\left(t_{i}\right)}\;\left[-\right]\\ E_{\text{surfactant}}\left(t_{i}\right)\;=\;\frac{c_{\text{surfactant},\text{ foamate}}\;\left(t_{i}\right)}{c_{\text{surfactant},\text{ reactor}}\;\left(t_{i}\right)}\;\left[-\right] \end{array}$$

### Rhamnolipid Purification

The purification of the rhamnolipids in the supernatant was performed by adsorption of the surfactants from a cell-free solution with a C18 derivatized silica-based adsorbent (AA12SA5, YMC Europe GmbH, Dinslaken, Germany). For desorption, pure ethanol was used as eluent. The eluate was evaporated and chromatographically separated using a preparative HPLC system consisting of an AZURA analytical pump P 6.1L, an AZURA autosampler 3950 (both Knauer GmbH, Berlin, Germany) connected to a SEDEX 58 LT-ELSD detector (SEDERE, Olivet, France), the fraction collector Foxy R1 (Teledyne ISCO Inc., Lincoln, NE, USA) and equipped with a VP250/21 NUCLEODUR C18 HTec column (particle size: 5 μm, Macherey-Nagel GmbH & Co. KG, Düren, Germany). The flow rate was set to 10 mL/min and 3 mL sample were injected. As eluent, acetonitrile and ultra-pure water supplemented with 0.2% (v/v) formic acid were used. The gradient was increased from 70 to 76% between 5 and 10 min, from 76 to 80% between 10 and 25 min, and to 100% until 35 min. It was decreased back to 70% between 45 and 50 min. Rhamnolipids were fractionated in between 25 and 47 min retention time. These fractions were evaporated to obtain pure, solvent-free rhamnolipids.

## Results

### Low Biomass Concentrations in Flask Cultivations Limits Production

**Figure 3:**
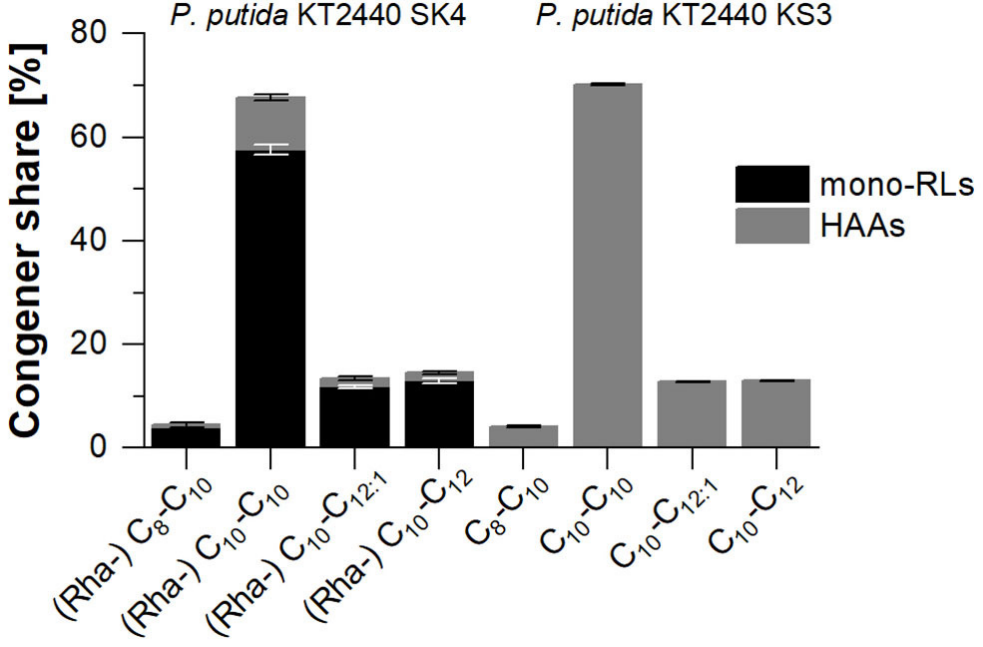
Congener composition of produced mono-rhamnolipids (black) and HAAs (gray) with *P. putida* KT2440 SK4 (left) and *P. putida* KT2440 KS3 (right) on minimal medium with glucose as sole carbon source. The error bars indicate the deviation from the mean of two biological replicates.

**Figure 4:**
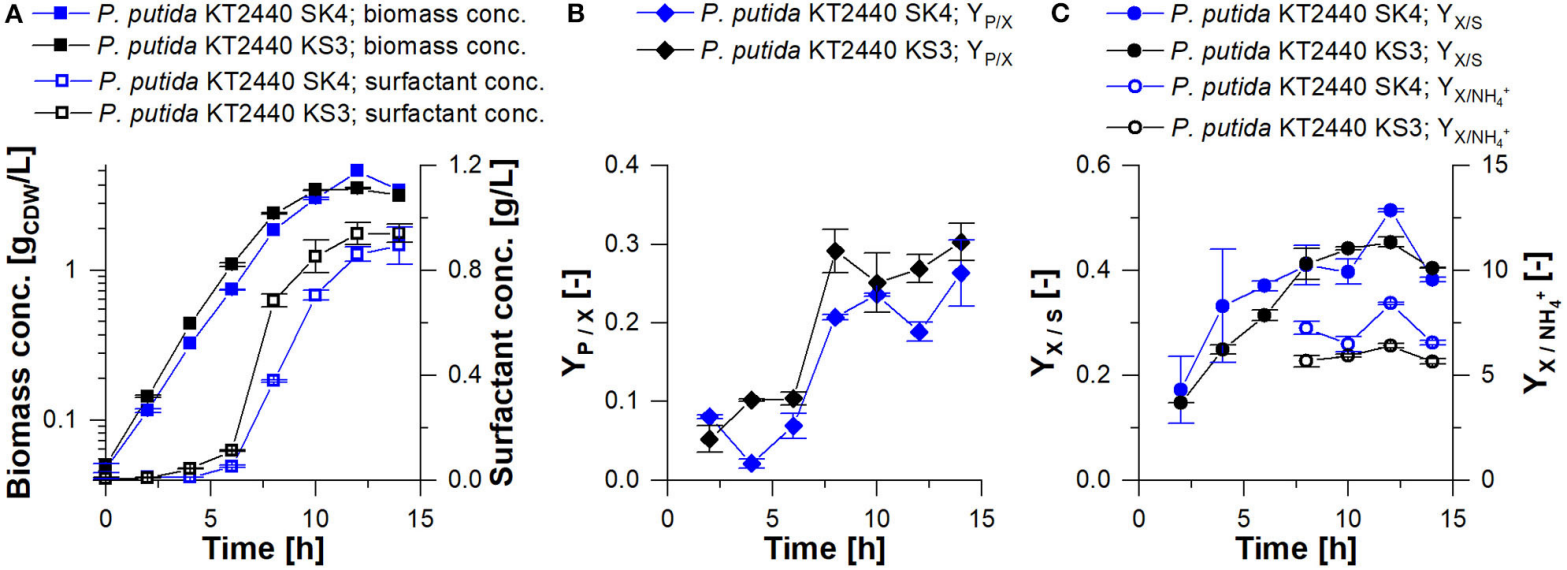
Shake flask cultivation of *P. putida* KT2440 SK4 (blue) and *P. putida* KT2440 KS3 (black) on minimal medium with 9 g/L glucose. **(A)** Biomass concentration vs. time (squares) and surfactant concentration vs. time (empty squares); **(B)** surfactant yield from biomass vs. time (*Y*_*P*/*X*_); **(C)** biomass yield from glucose vs. time (*Y*_*X*/*S*_, points) and biomass per ammonium vs. time ($Y_{X/{\text{NH}}_{4}^{+}}$, empty points). The error bars indicate the deviation from the mean of two biological replicates.

In shake flask cultivations, the rhamnolipid production strain *P. putida* KT2440 SK4 and the HAA production strain *P. putida* KT2440 KS3 reached final titers of 0.91 ± 0.14 g_RL_/L and 0.94 ± 0.07 g_HAA_/L, respectively (**Figure 4**A). For *P. putida* KT2440 SK4, as for all rhamnolipid producers in this study, the produced rhamnolipid concentration is defined as the sum of synthesized HAAs and mono-rhamnolipids. The congener compositions of synthesized rhamnolipids and HAAs are depicted in Figure 3. For both strains, the C_10_-C_10_ dimer is dominant with a share of over 60% (w/w).

In the applied shake flask cultivations, foam formation occurred only marginally due to laminar fluid motion, ensuring homogeneous conditions. For *P. putida* KT2440 SK4 and KS3, the maximal growth rates were 0.46 ± 0.02 h^−1^ and 0.48 ± 0.003 h^−1^, respectively (Table 2). The highest surfactant production rate was detected in the late exponential phase after 6 h (Figure 4). Until carbon depletion, total space-time yields of *STY*_SK4, flask_= 0.06 ± 0.01 g_RL_/L·h and *STY*_KS3, flask_= 0.07 ± 0.01 g_HAA_/L·h were reached. By referencing product formation to substrate, final yields were similar at *Y*_*P*/*S*_= 0.1 g_*P*_/g_*S*_ (Table 2). For increased space-time yield for HAA and rhamnolipid production, higher biomass concentrations in the culture are however necessary. A bioreactor process is designed according to the identified need for carbon and nitrogen.

**Table 2:** Performance indicators of the two engineered recombinant biosurfactant producers in shake flask cultivations.

***P. putida* KT2440 strain**	**Product**	****μ****_**max**_ **[1/h]**	***STY* [g_***P***_/L·h]**	***Y*_*P*/*X*_** **[g**_***P***_**/g**_***X*]**_	***Y*_*P*/*S*_** **[g**_***P***_**/g**_***S***_**]**	***Y*_*X*/*S*_** **[g**_***X***_**/g**_***S***_**]**	***Y*_*P*/*X*_** **[g**_***X***_**/g**${\;}_{{\text{NH}}_{4}^{+}}$**]**
SK4	RL	0.46 ± 0.02	0.06 ± 0.01	0.26 ± 0.04	0.1 ± 0.02	0.38 ± 0.00	6.55 ± 0.1
KS3	HAA	0.48 ± 0.003	0.07 ± 0.01	0.3 ± 0.02	0.12 ± 0.01	0.4 ± 0.0	5.66 ± 0.12

*Biological replicates (n = 2) with deviation from the mean are shown*.

Latest published HAA syntheses with a plasmid-based production host reached space-time yields of 0.06 g_HAA_/L·h (Germer et al., 2020) and 0.07 g_HAA_/L·h (Tiso et al., 2017) in complex medium. Now, by integrating the production cassette into the genome for the construction of *P. putida* KT2440 KS3, a stable HAA producing strain is available, which does not require the addition of antibiotics. Especially concerning applications in larger scales, the renunciation of antibiotics reduces costs significantly. With the same STY as the previously mentioned strains even on minimal medium (0.07 g_HAA_/L·h), *P. putida* KT2440 KS3 has great potential for further applications regarding HAA synthesis.

### Bioreactor Cultivations Lead to Increased Space-Time Yield

**Figure 5:**
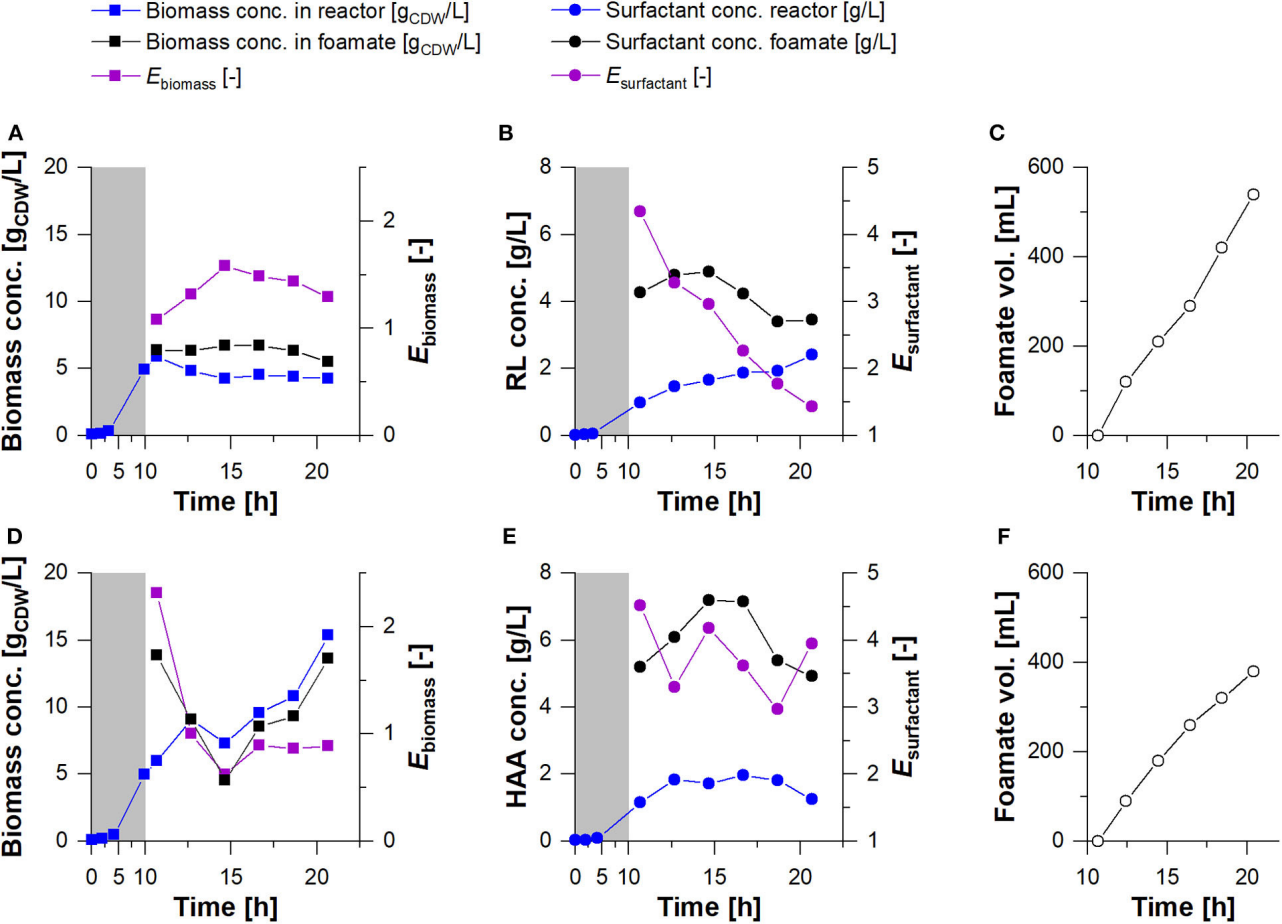
Cultivation of *P. putida* KT2440 SK4 **(A–C)** and *P. putida* KT2440 KS3 **(D–F)** in a bioreactor. Growth phase (gray background) and a subsequent continuous product separation during the harvest phase (*t* = 10.6 h to *t* = 20.6 h). **(A,D)** Biomass concentration in the reactor (blue squares) and in the foamate (black squares) and **(B,E)** Surfactant concentrations were measured in the fermentation broth of the reactor (blue points) and in the foamate after fractionation (black points). The biomass and surfactant enrichment factors (*E*_biomass_ & *E*_surfactant_) are depicted in violet. **(C,F)** The total volume of separated foamate was measured continuously.

As higher biomass concentrations, and consequently higher concentrations of biocatalysts are mandatory for an improved HAA and rhamnolipid production without any limitations in substrates and oxygen, a bioreactor cultivation process was designed. In the bioreactor setup (Figure 2), the process was divided into a growth and a harvest phase: 1. In the growth phase, the foam leaving the gas exhaust of the reactor was collapsed and continuously recirculated into the reactor. This prevented the loss of biomass by cells entrapped in the foam and resulted in biomass concentrations of 5 g_CDW_/L and growth rates of μ= 0.44 h^−1^ for both producer strains. 2. In the harvest phase conducted for 10 h, the fractionated foam was collected as foamate. Here, biomass concentration in the reactor could be maintained for *P. putida* KT2440 SK4, while the biomass concentration of *P. putida* KT2440 KS3 increased continuously (Figures 5A,D). The biomass concentrations in the foamate, leaving the foam centrifuge showed the same trends. However, in comparison to the rhamnolipid producer, the HAA producing strain showed slightly lower biomass concentrations in the foamate than in the culture broth. The correlation of biomass in foamate to reactor is indicated as enrichment factor *E*_biomass_ (Figures 5A,D). Here, at values above 1, the cells were enriched in the foamate. This was observed during rhamnolipid production over the entire cultivation time, while in the HAA fermentation, the enrichment was high at the beginning (*E*_biomass_> 2) and ends at *E*_biomass_ = 0.9. The surfactant enrichment via foam fractionation enabled rhamnolipid concentrations over 4 g_RL_/L and HAA concentrations higher than 7 g_HAA_/L (Figures 5B,E) in the foamate. In total, 540 mL foamate with an overall rhamnolipid concentration of about 4 g_RL_/L were separated (Figure 5C). Together with the product that remained in the reactor, 7 g rhamnolipids were produced. That results in *STY*_SK4, reactor_ = 0.17 g_RL_/L·h. With *P. putida* KT2440 KS3, HAA concentrations in the reactor stayed stable while the biomass concentration increased, indicating an efficient product separation. Contrary to the rhamnolipid fractionation, the volume of the foamate in the collection bottle increased slower after the surfactant concentration in the foamate has dropped (Figure 5F). Here, it is very likely that HAAs were degraded in the column by the increasing presence of biomass. High biodegradability of HAAs, while rhamnolipids remain non-degraded, was already discussed by Tiso et al. (2017). With a lower surfactant concentration, less foam could be collected in the foamate vessel. However, a constantly higher HAA enrichment (*E*_HAA, mean_ = 3.75 ± 0.5) compared to the rhamnolipid enrichment (*E*_RL, mean_ = 2.68 ± 0.98) was apparent (Table 3). With the absence of the polar rhamnose residue, HAAs feature a lowered amphiphilicity compared to rhamnolipids. Consequently, the tendency to accumulate at the gas-liquid interface is lower, thus reducing the intensity of foam formation (Tiso et al., 2017). The average foamate flow of ${\overset{∙}{V}}_{\text{HAA},\text{ foamate }}=$ 38 mL/h, was 1.4 times lower than the rhamnolipid foamate flow. Potentially, the liquid content of HAA foam was lower when it left the reactor, causing even higher enrichments after the fractionation. For HAA production, 380 mL foamate with a concentration of 6 g_HAA_/L were harvested and 2.5 g_HAA_/L remained in the reactor, leading to a total process *STY*_KS3, reactor_ = 0.12 g_HAA_/L·h. Compared to the rhamnolipid and HAA synthesis in the shake flasks, the STY was increased by 2.8-fold and by 1.7-fold, respectively. This improvement is achieved despite the loss of cells from the reactor into the fractionated foam.

**Table 3:** Process parameters of bioreactor cultivations for rhamnolipid or HAA production and recovery.

***P. putida* KT2440 strain**	**KS3**	**Δ*lapF*_HAA**	**SK4**	**Δ*lapF*_RL**	**Δ*lapA* Δ*lapF*_RL**	**Δflag_RL**	**GR20_RL**
Product	HAA	HAA	RL	RL	RL	RL	RL
Synthesized product [g]	4.8	2.8	7.1	4.6	8.1	8.7	10.0
Separated product [%]	48.2	50.5	32.1	36.4	30.0	28.3	31.3
*E*${\;}_{\text{biomass}}^{*}$ [–]	1.1 ± 0.6	0.73 ± 0.2	1.36 ± 0.2	1.14 ± 0.2	1.27 ± 0.3	0.74 ± 0.23	0.82 ± 0.3
*E*${\;}_{\text{surfactant}}^{*}$ [–]	3.75 ± 0.5	4.98 ± 1.6	2.68 ± 1.0	4.3 ± 1.2	2.47 ± 0.3	2.23 ± 0.4	1.69 ± 0.3
*Y*_*P*/*S*_ [–]	0.04	0.02	0.07	0.04	0.07	0.08	0.09
*STY* [g_*P*_/L·h]	0.12	0.07	0.17	0.11	0.19	0.21	0.24

^*^Mean for all determined enrichment factors E with standard deviation during harvest period (n = 6).

### Surface Structures Have a Significant Influence on CSH and Thus Bacterial Foam Adhesion

**Figure 6:**
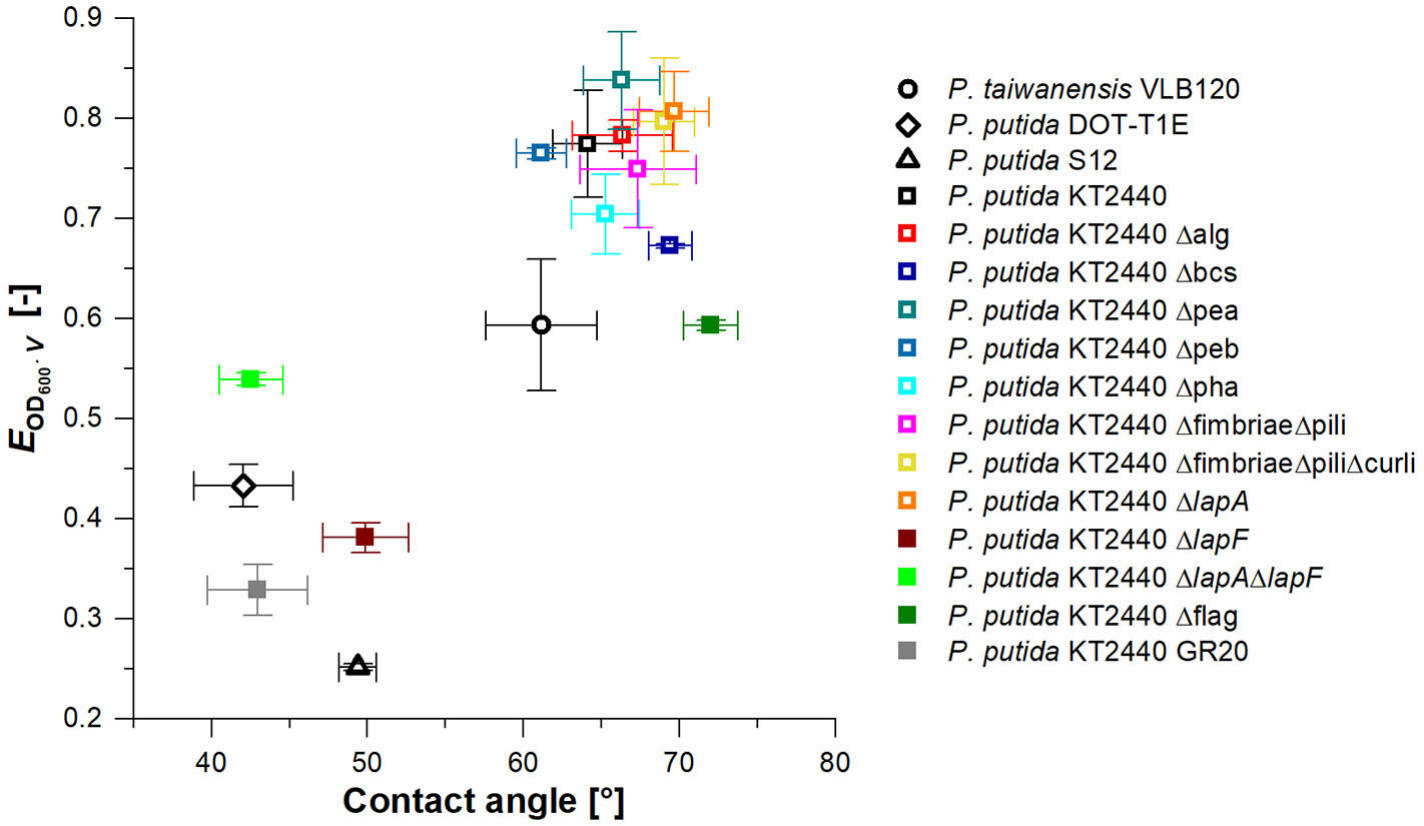
Biomass enrichment factor *E*_*O*_*D*__600_·*V*_ (*n* = 2) in fractionated foam vs. water contact angle (*n* = 9) for *Pseudomonas* wild types (black) and *P. putida* KT2440 knock-out strains. *P. putida* KT2440 knock-out strains used for *rhlA* and *rhlAB* integration in the following are marked by filled squares. Vertical error bars indicate the deviation from the mean for *n* = 2 and horizontal error bars the standard deviation of the mean for *n* = 9.

In the previous fermentation, high biomass loss due to foam adhesion of cells was observed. In addition to reduced productivity, higher biomass concentrations in the foamate result in a more complex downstream processing (DSP). Consequently, *P. putida* KT2440 was modified to identify cell surface structures responsible for cell surface hydrophobicity (CSH), thereby contributing to foam adhesion. A broad range of surface structures was removed by genetic engineering. By foaming a rhamnolipid solution with added surface-modified strains vertically through a column, foam adhesion of these strains was quantified. The introduced enrichment factor *E*_*O*_*D*__600_·*V*_ was used here to assess the foam adhesion tendency of the individual strains. More than half of the investigated strains had an *E*_*O*_*D*__600_·*V*_ of 0.65 to 0.85, corresponding to the highest observed values (Figure 6). With *E*_*O*_*D*__600_·*V*_ = 0.77, the enrichment factor of the *P. putida* KT2440 wild-type strain is located in this range together with strains with a deleted synthesis of exopolysaccharide a and b (Δpea & Δpeb), alginate (Δalg), as well as fimbriae and pili (ΔfimbriaeΔpili). Therefore, these surface structures seemed to have no significant impact on bacterial foam adhesion. *P. putida* KT2440 without the flagellum (Δflag), the adhesin LapF (Δ*lapF*), and LapA and LapF combined (Δ*lapA*Δ*lapF*) depict *E*_*O*_*D*__600_·*V*_ values of below 0.65. With the multi-deletion-strain *P. putida* KT2440 GR20, the lowest tendency for foam adhesion for knock-out mutants was reached with an *E*_*O*_*D*__600_·*V*, GR20_ of 0.33. *P. putida* KT2440 GR20 contains among other deletions, no flagellum and no LapA and LapF. Notably, the minimal cell enrichment in the foam was reached with a *P. putida* S12 wild-type strain.

In order to validate the impact of the applied deletions on the CSH, water contact angles of the correspondent bacterial lawn were recorded. To evaluate if a correlation between CSH and foam adhesion existed, the CSH values were plotted against the biomass enrichment in the foam (Figure 6). In general, the same trend was observed as seen for the enrichment test. Many mutations did not influence the CSH having contact angles between 60 and 72° like *P. putida* KT2440. *P. putida* KT2440 Δflag, a strain among those with a reduced *E*_*O*_*D*__600_·*V*_ value, was also within the water contact angle range of the wild-type strain. A reason for this phenomenon is probably the relatively low size of the flagellum compared to the total cell surface and therefore a low influence on CSH. The impact on enrichment might be caused by its long hydrophobic tail, acting like an anchor in the hydrophobic air bubbles. Apart from the flagellum deletion, mutants featuring a lower foam adhesion tendency also demonstrated a lower surface hydrophobicity based on a water contact angle of 40 to 50°. These results indicate that the factor *E*_*O*_*D*__600_·*V*_ correlates to the CSH. The knock-out mutants standing out (i.e., which are in the lower left quadrant of the graph) were *P. putida* KT2440 Δ*lapF, P. putida* KT2440 Δ*lapA*Δ*lapF*, and the cumulative deletion-strain *P. putida* KT2440 GR20. Additionally, wild-type strains *P. putida* DOT-T1E and S12 are among the best performing strains. According to a BLAST analysis (Altschul et al., 1990), *P. putida* DOT-T1E, and S12 genomes contain no gene encoding for the adhesin LapF. These strain-to-strain differences explain why many *P. putida* strains are in use and still new isolates with additional phenotypes are reported.

### Enhanced Product Separation From Biomass With Cell Surface-Modified Biocatalysts

**Figure 7:**
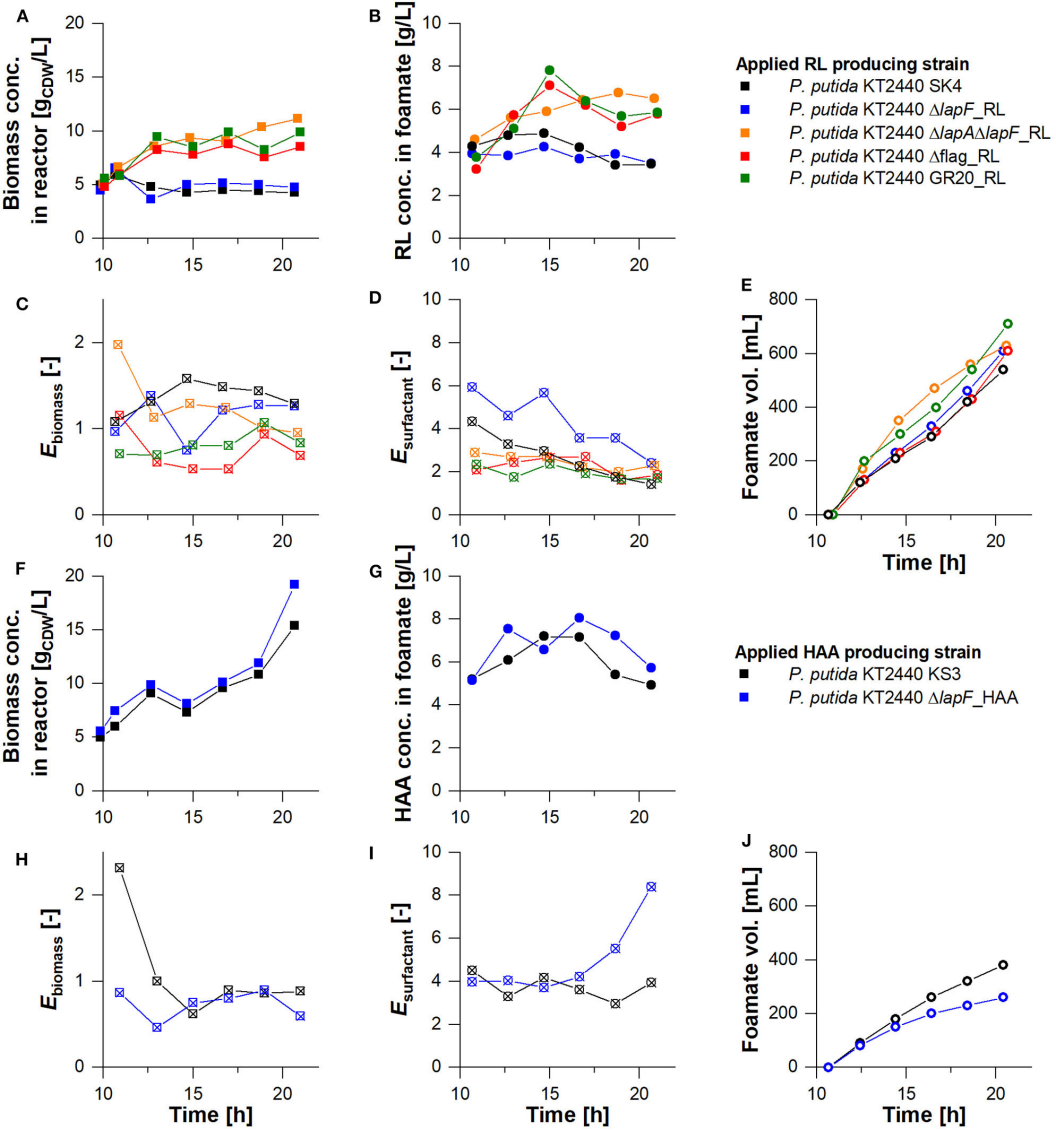
Comparison of non-modified production hosts (black) with knock-out strains negative for the synthesis of surface structures (colored). Harvest phase at the **(A–E)** rhamnolipid and **(F–J)** HAA production and separation as foamate in the designed bioreactor setup. **(A,F)** Biomass concentration in the reactor vs. time, **(B,G)** biosurfactant concentration in the foamate at the inlet of the collection bottle vs. time, **(C,D,H,I)** biomass and surfactant enrichment in the foamate (*E*_biomass_ & *E*_surfactant_) vs. time, and **(E**, **J)** the volume of the collected foamate vs. time.

The strains that featured a lower foam adhesion were equipped with rhamnolipid and HAA production genes and used for fermentation. The aim was to show that a low biomass accumulation in the foam could be achieved not only in stand-alone tests but also in the actual production process. To focus on the agglomeration tendency of strains in the foam, all process conditions were kept exactly as applied for the non-modified production strains earlier. The biomass concentrations of surface-modified strains exclusively for the harvest phase, are depicted in Figures 7A,F, with the non-modified strains as reference. Except for *P. putida* KT2440 Δ*lapF*_RL, all biomass concentrations in the reactor and the rhamnolipid concentration in the foamate rose. *P. putida* KT2440 Δflag_RL and *P. putida* KT2440 GR20_RL cultures reached biomass concentrations higher than 9 g_CDW_/L and caused the highest rhamnolipid concentrations in the separated foamate, with values over 7 g_RL_/L. For all surface-modified HAA and rhamnolipid production hosts, biomass enrichment factors *E*_biomass_ were on average lower than the enrichments measured for *P. putida* KT2440_RL (*E*_biomass_= 1.36 ± 0.2) and *P. putida* KT2440 KS3 (*E*_biomass_= 1.1 ± 0.6). *P. putida* KT2440 Δflag_RL and *P. putida* KT2440 GR20_RL had minimal average biomass flotation tendencies with *E*_biomass_= 0.74 ± 0.23 and *E*_biomass_= 0.82 ± 0.3, respectively (Figure 7C, Table 3). With *P. putida* KT2440 Δflag_RL, the bacterial foam adhesion could be reduced by 46%. In contrast to the stand-alone bacterial foam adhesion tests, the strain without flagellum enriched less in the foam than strains with deleted genes encoding for LapA and LapF.

In terms of surfactant enrichment, the average rhamnolipid enrichment by fractionation lay between *E*_surfactant_= 1.69 ± 0.3 and 2.68 ± 1 except for the cultivation of *P. putida* KT2440 Δ*lapF*_RL (Table 3). The rhamnolipid enrichment dropped throughout the harvest phase in all experiments. As already shown with the HAA production host without surface modifications, biomass foam adhesion was generally lower and product enrichment was higher than in rhamnolipid synthesizing processes. These characteristics, which promote the separation process, were confirmed with the HAA production strain without LapF. *P. putida* KT2440 Δ*lapF*_HAA continuously grew in the harvest phase, reaching a final concentration of 19 g_CDW_/L (Figure 7F). The HAA concentration trend in the foamate showed the same curve as the HAA concentration trend of the non-modified strain, reaching 8 g_HAA_/L (Figure 7G). With the *lapF* deletion, the biomass enrichment in the foamate was on average lower and the surfactant enrichment higher. A product enrichment factor of *E*_surfactant_= 4.98 ± 1.6 made *P. putida* KT2440 Δ*lapF*_HAA the strain with the highest *E*_surfactant_ value.

Besides the investigated biomass agglomerations and product enrichments in the foam, the optimized bioreactor process had to be analyzed concerning biosurfactant productivity and product separation efficiency. With 10 g produced rhamnolipids, the cumulative deletion-strain *P. putida* KT2440 GR20_RL was the most efficient producer (Table 3). Consequently, the reached *STY*_GR20_RL_ = 0.24 g_RL_/L·h was 1.4-fold improved compared to the space-time yield that has been achieved with the rhamnolipid producer without surface modifications. Higher surfactant concentrations provoked a fortified foam formation. *P. putida* KT2440 GR20_RL produced 1.3-fold more foamate in the collection bottle than using the second-best rhamnolipid producer *P. putida* KT2440 Δflag_RL (Figure 7E). In general, 28 to 36% of the total produced rhamnolipids were separated via foam fractionation. Again, higher separation efficiencies could be realized in the HAA production process. For both applied strains, *P. putida* KT2440 KS3 and *P. putida* KT2440 Δ*lapF*_HAA, about half of the secreted HAAs were transferred into the foamate collection bottle. Despite a lowered biomass enrichment of 34% in the foamate with a LapF negative strain, no higher productivity could be obtained in comparison to *P. putida* KT2440 KS3.

## Discussion

### Integrated Foam Fractionation as a Trade-Off Between Productivity and Separation Efficiency

The applied bioreactor setup with an integrated foam fractionation achieved high STYs for rhamnolipid and HAA production. However, this high productivity was achieved at the expense of lower product separation efficiency, as at least half of the total produced surfactant remained in the culture broth. Beuker et al. (2016b) used a setup with integrated foam fractionation similar to the one presented in this study. A lower biomass concentration of 3.3 g_CDW_/L, compared to 5 g_CDW_/L in our experiments was reached after a cultivation time of 10 h. A lower growth is most likely caused by the lower gassing rate of 0.067 vvm compared to the applied gassing rate of 0.25 vvm in our experiments, as growth of the aerobic *P. putida* is impaired when not enough oxygen is available. Furthermore, foam separation was conducted right from the beginning of the fermentation, most likely reducing biomass amounts in the liquid in the study of Beuker et al. (2016b). In a similar setup Anic et al. (2018) applied a gassing rate of 0.1 vvm and installed an additional foamate reflux, reaching a biomass concentration of more than 5 g_CDW_/L after 40 h. Foam destabilization was carried out by an integrated rhamnolipid adsorption. In our study, in the harvest phase, the fractionation efficiency declines after 5 h. With *P. putida* KT2440 SK4, the rhamnolipid concentration in the reactor broth rose, resulting in a lowered enrichment factor. Higher rhamnolipid concentrations in the broth led to wetter foam. This is underlined by an increased rate of foamate formation over time while surfactant concentrations in the foamate declined (Figure 7), a phenomenon also reported by Anic et al. (2018). The operation window for optimal foam fractionation is a trade-off. On the one hand, higher gassing rates omit oxygen limitations in the broth promoting rapid microbial growth and surfactant production, whereas an efficient product separation is achieved by reducing gassing rates. With the here developed multi-stage process (divided into growth and harvest phase), a 4.5 and a 2.3-fold higher STY could be achieved for rhamnolipid production by *P. putida* KT2440 SK4 with continuous foam fractionation compared to Beuker et al. (2016b) and Anic et al. (2018), respectively. Even though the product recovery in the foam of 97% reported from Beuker et al. (2016b) is much higher than in this study. Here, only 32% of the produced rhamnolipids were separated by foam fractionation. Probably during higher aeration, the liquid content in the foam increases, changing also the content of biosurfactant in the foamate. To avoid the dependence of separation efficiency on the gassing rate, immobilized cells can be applied, e.g., by entrapment of microbial cell factories in polymers (Siemann and Wagner, 1993; Heyd et al., 2011). With no cells leaving the reactor, foam fractionation conditions can be optimized, e.g., by an increased residence time in the fractionation column with larger column dimensions (Sarachat et al., 2010). As the quantitative surfactant secretion into the medium depends on culture conditions as cell vitality, growth, and density, the regulation of these conditions is of central importance. However, as already discussed for the gassing rate, process variables have a direct impact on the subsequent fractionation.

### Abiotic Parameters Reveal Potential for Increasing the Process Efficiency

**Figure 8:**
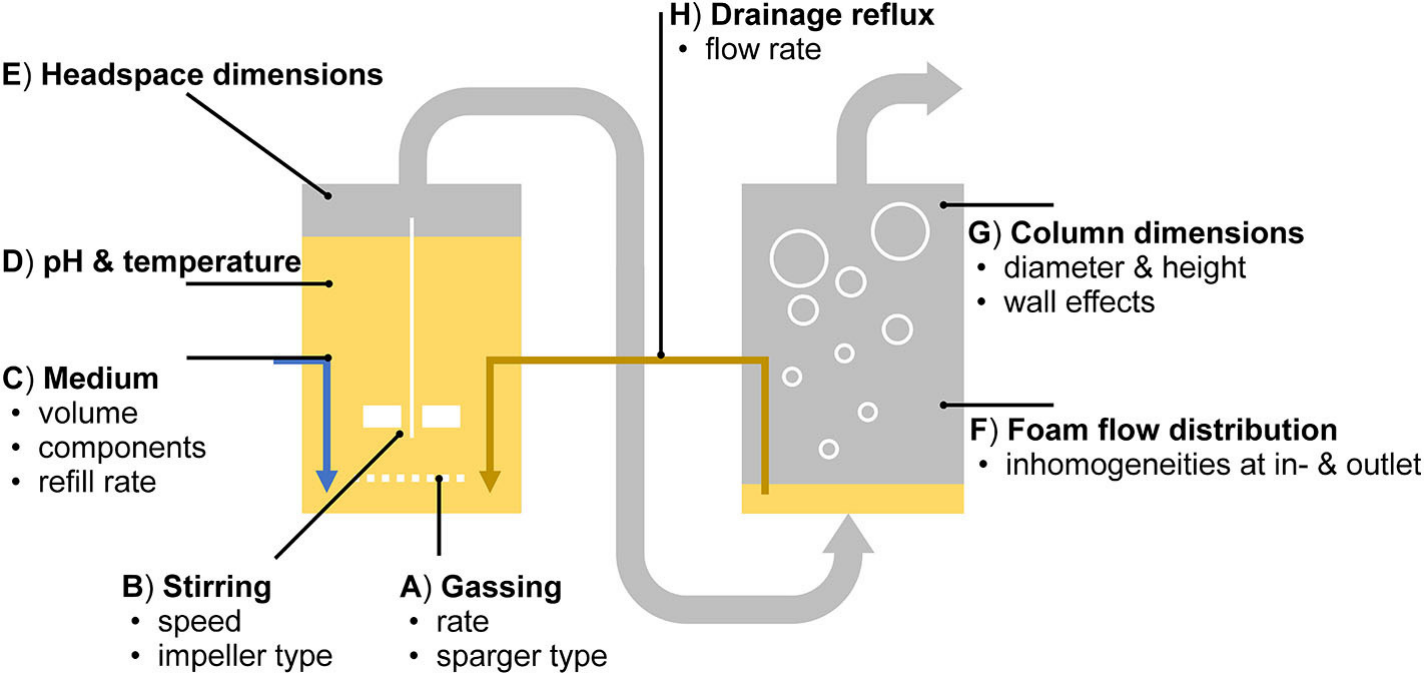
Abiotic influence factors on biosurfactant production in the applied aerated bioreactor system with product separation via integrated foam fractionation.

In the applied setup, technical adjustments for enhanced process efficiency are numerous (Figure 8). They are briefly outlined here in the context of studies focused on individual process variables. (A) The gas-liquid surface area is dependent on the bubble size, which can be adjusted by altering the diameters of the pores in the sparger while maintaining the gassing rate (Khanchezar et al., 2019). (B) The stirring speed influences the water content in the foam (Long et al., 2016). (C) Medium components, such as multivalent anionic ions (e.g., Mg^2+^) are discussed to reduce bacterial flotation (Somasundaran, 1975; Beuker et al., 2016b). (D) With a lowered pH, rhamnolipids form less foam (Özdemir et al., 2004). (E) In this work, a rather small headspace volume was chosen to guarantee stable foaming through the reactor outlet, even at low surfactant concentrations. (F) For the headspace, as for the connected foam fractionation column, the vertical flow behavior is intended to be as homogeneous as possible, which is especially challenging at the in- and outlets. (G) With increased column dimensions at a constant height to diameter ratio, separation efficiencies increase due to a lower impact of wall effects (Merz, 2012). Again, the conditions in the reactor are subjected to change by a correspondent (H) return of medium and biocatalysts impacting the cultivation. Despite the many parameters influencing the process performance, the here developed setup facilitated the identification of a suitable set of parameters for stable process operation.

### Cell Surface-Modified Strains for Enhanced Production

While metabolic traits are often targets for strain improvement, cell surface properties are rarely engineered. Anic et al. (2017) suggested that *P. putida* without the flagellar machinery has a reduced tendency to agglomerate in foam, which was confirmed here. Despite that, we did not observe a lowered CSH. However, Martinez-Garcia et al. (2014) measured a lower CSH of a *P. putida* KT2440 flagellum deletion strain in comparison to the wild type via a microbial adherence to hydrocarbon (MATH) test (Rosenberg et al., 1980). Furthermore, the same study showed that non-flagellated cells form more biofilm than wild-type cells, fostered by a de-repression of exopolysaccharide production (Martinez-Garcia et al., 2014). This finding could be the reason why the lowest biomass agglomeration in foam was detected for the cumulative deletion-strain *P. putida* KT2440 GR20, which furthermore features EPS- and flagellum deletions. Other studies confirmed a reduced bacterial CSH by flagellum removal in *P. aeruginosa* (Bruzaud et al., 2015) and *E. coli* (Friedlander et al., 2015) strains. An interesting finding is the low foam adhesion by *P. putida* S12 and *P. putida* DOT-T1E. While for *P. putida*, it is known that the adhesin LapF increases CSH (Lahesaare et al., 2016), no homolog of *lapF* was found on the respective genomes of these strains. However, at least in Germany, all *P. putida* strains except KT2440 are of biosafety level 2 (Nelson et al., 2002; ZKBS, 2012), a true challenge for the development of new bioprocesses, strongly advocating the usage of *P. putida* KT2440.

By using CSH mutants with lower foam enrichment, the outcomes of the stand-alone tests were confirmed. Again, all strains showed reduced enrichment, even though the reduction occurred in a different order. In the reactor experiments, the *lapF* deletion affected biomass adhesion in the foam less than the flagellum deletion. Also, the cumulative deletion-strain *P. putida* KT2440 GR20_RL with the lowest enrichment in the biomass flotation tests had a higher enrichment than *P. putida* KT2440 Δflag_RL in the foamate during bioreactor cultivation (Table 3). In other studies is was reported that flagellum deletion improves biomass yield on substrate (Martinez-Garcia et al., 2014) as well as the rhamnolipid production performance of the microbial cell factory (Tiso et al., 2020a). Higher biosurfactant production influences foaming and therefore the biomass adhesion tendency as well. The assessment of the biomass agglomeration promoted by certain surface structures with the performed stand-alone biomass flotation experiments that were always conducted with the same surfactant concentration therefore provides a better insight into the monocausal relation between CSH and cell foam adhesion. However, the results from the fermentation experiments allow for a better assessment and selection of the strain best suited for the here developed process. Overall, *P. putida* KT2440 GR20_RL is the best producer, reaching almost 10 g rhamnolipids within 20 h. With *P. putida* KT2440 Δflag_RL, 8.7 g rhamnolipids were produced in total, resulting in a 1.2 times higher production than *P. putida* KT2440 SK4.

In summary, we could show that genetic modifications of the bacterial cell surface reduced foam adhesion. This reduction in cell adhesion allowed stable rhamnolipid and HAA production in aerated bioreactors without the need of, e.g., antifoam addition. The expected benefits are not only lower operation cost of biosurfactant production, but especially reduced cost in the subsequent DSP. The integration of strain and process engineering, as discussed by Kuhn et al. (2010), clearly opens new possibilities for tailored process designs (Singh et al., 2019).

## Data Availability Statement

All datasets generated for this study are included in the article/Supplementary Material.

## Author Contributions

CCB conducted the tests for biomass agglomeration in the foam and contact angle measurements for cell surface hydrophobicity detection. Furthermore, CCB performed all shake flask and bioreactor experiments. CCB analyzed the data, prepared the figures and wrote the manuscript. IB generated all *P. putida* KT2440 deletion-strains and most of the rhamnolipid producers. HJH and CE advised on contact angle measurements, gave support in all questions concerning cell surface hydrophobicity and revised the manuscript. TT and LMB initiated the project, advised on all experiments, analyzed and discussed data, and edited the manuscript. All authors read and approved the final manuscript.

## Conflict of Interest

LMB and TT declare that they are inventors of three related patents. (1) LMB, F. Rosenau, S. Wilhelm, A. Wittgens, TT, “Means and methods for rhamnolipid production” HHU Düsseldorf University, TU Dortmund University, 2013 (WO 2013/041670 A1), (2) LMB, B. Küpper, E. M. del Amor Villa, R. Wichmann, C. Nowacki, “Foam adsorption” TU Dortmund University, 2013 (WO 2013/087674 A1), and (3) LMB, TT, A. Germer, “Extracellular production of designer hydroxyalkanoyloxy alkanoic acids with recombinant bacteria” RWTH Aachen University, 2015 (WO2017006252A1). The remaining authors declare that the research was conducted in the absence of any commercial or financial relationships that could be construed as a potential conflict of interest.
