# Supplementary material for: Genetic Cell-Surface Modification for Optimized Foam Fractionation
Source: Front Bioeng Biotechnol. 2020 Oct 29;8:572892. doi: 10.3389/fbioe.2020.572892 (PMC7658403; doi:10.3389/fbioe.2020.572892)
Supplement: Supplementary file 1 [file Data_Sheet_1.doc]

Supplementary Material

Genetic cell-surface modification for optimized foam fractionation

Christian C. Blesken^1^, Isabel Bator^1,2^, Christian Eberlein^3^, Hermann J. Heipieper^3^, Till Tiso^1,2^^*^, Lars M. Blank^1,2*^

^1^ iAMB - Institute of Applied Microbiology, ABBt - Aachen Biology and Biotechnology, RWTH Aachen University, Aachen, Germany

^2^ Bioeconomy Science Center (BioSC), Forschungszentrum Jülich GmbH, Jülich, Germany

^3^ UFZ - Helmholtz Centre for Environmental Research, Department of Environmental Biotechnology, Leipzig, Germany

*** Correspondence:** Till Tiso & Lars M. Blank
[till.tiso@rwth-aachen.de](mailto:till.tiso@rwth-aachen.de) & [lars.blank@rwth-aachen.de](mailto:lars.blank@rwth-aachen.de)

# Supplementary Figures and Tables

## Supplementary Figures

| 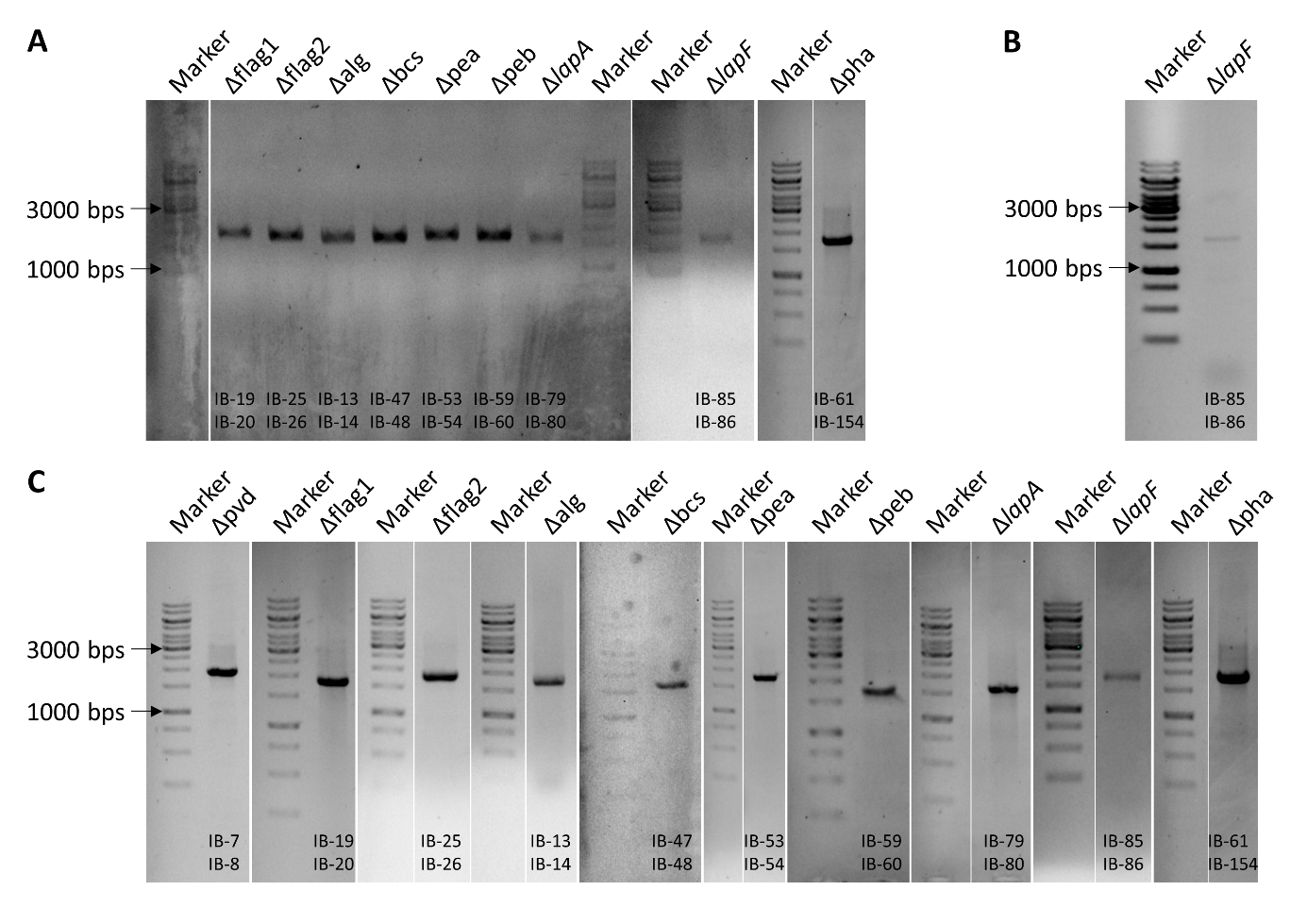 |
| --- |
| **Figure 1.** Knock-out verification by colony PCR. (A) Agarose gel electrophoresis of products of colony PCRs to verify the deletion of the different genomic regions from *P. putida* KT2440. (B) Agarose gel electrophoresis of colony PCR to verify the deletion of *lapF* from *P. putida* KT2440 Δ*lapA*. (C) Agarose gel electrophoresis of colony PCRs to verify the deletion of the different genomic regions in the cumulative *P. putida* KT2440 GR20 strain. As marker the GeneRuler 1 kb DNA Ladder (Thermo Fisher Scientific, Waltham, MA, USA) was used. The used oligonucleotides are indicated below the bands and for each amplification a DNA fragment between 1,500 and 2,000 was expected as the oligonucleotides bind upstream and downstream of the flanking TS-regions |

## Supplementary Tables

Supplementary Table 1. List of oligonucleotides used in this work.

| Name | Direction | Used for | Sequence |
| --- | --- | --- | --- |
| KS02  KS08  IB-5  IB-6  IB-1  IB-2  IB-3  IB-4  IB-7  IB-8  IB-9  IB-10  IB-11  IB-12  IB-13  IB-14  IB-15  IB-16  IB-17  IB-18  IB-19  IB-20  IB-21  IB-22  IB-23  IB-24  IB-25  IB-26  IB-43  IB-44  IB-45  IB-46  IB-47  IB-48  IB-49  IB-50  IB-51  IB-52  IB-53  IB-54  IB-55  IB-56  IB-57  IB-58  IB-59  IB-60  IB-61  IB-154  IB-75  IB-76  IB-77  IB-78  IB-79  IB-80  IB-81  IB-82  IB-83  IB-84  IB-85  IB-86 | rev  fwd  fwd  rev  fwd  rev  fwd  rev  fwd  rev  fwd  rev  fwd  rev  fwd  rev  fwd  rev  fwd  rev  fwd  rev  fwd  rev  fwd  rev  fwd  rev  fwd  rev  fwd  rev  fwd  rev  fwd  rev  fwd  rev  fwd  rev  fwd  rev  fwd  rev  fwd  rev  fwd  rev  fwd  rev  fwd  rev  fwd  rev  fwd  rev  fwd  rev  fwd  rev | construction of pKS03  construction of pKS03  pEMG-plasmids  pEMG-plasmids  pvd-TS1 amplification  pvd-TS1 amplification  pvd-TS2 amplification  pvd-TS2 amplification  pvd-KO verification  pvd-KO verification  alg-TS1 amplification  alg-TS1 amplification  alg-TS2 amplification  alg-TS2 amplification  alg-KO verification  alg-KO verification  flag1-TS1 amplification  flag1-TS1 amplification  flag1-TS2 amplification  flag1-TS2 amplification  flag1-KO verification  flag1-KO verification  flag2-TS1 amplification  flag2-TS1 amplification  flag2-TS2 amplification  flag2-TS2 amplification  flag2-KO verification  flag2-KO verification  bcs-TS1 amplification  bcs-TS1 amplification  bcs-TS2 amplification  bcs-TS2 amplification  bcs-KO verification  bcs-KO verification  pea-TS1 amplification  pea-TS1 amplification  pea-TS2 amplification  pea-TS2 amplification  pea-KO verification  pea-KO verification  peb-TS1 amplification  peb-TS1 amplification  peb-TS2 amplification  peb-TS2 amplification  peb-KO verification  peb-KO verification  pha-KO verification  pha-KO verification  lapA-TS1 amplification  lapA-TS1 amplification  lapA-TS2 amplification  lapA-TS2 amplification  lapA-KO verification  lapA-KO verification  lapF-TS1 amplification  lapF-TS1 amplification  lapF-TS2 amplification  lapF-TS2 amplification  lapF-KO verification  lapF-KO verification | TCTTCGCAGGTCAAGGGTTC  gaacccttgacctgcgaagaGAATTCGAGCTCGGTACCCG  CAAGGCGATTAAGTTGGG  TCCGGCTCGTATGTTGTG  taacagggtaatctgaattcGCAGGTGCACTGTTGCTGGC  gaccccgagcGCTTTCGGGGCCGCCAGC  ccccgaaagcGCTCGGGGTCCTTACTGATC  ttgcatgcctgcaggtcgacTACAGCCTCGACTGGCCG  ACCCATACGCATGAAGTC  TACTGCTGCGTGGTTTCG  taacagggtaatctgaattcCACCAGCCAGGTGAGGATC  ggtgatagcgATGCTGACTCGCCCCTGG  gagtcagcatCGCTATCACCTCATGTGTTTATC  ttgcatgcctgcaggtcgacCGAATCCGACGCCAAAAATC  TCTTGCCAGACCACGAAC  TACTACAGTGCCGAGCAG  taacagggtaatctgaattcGCTGCCAGGTGCGCTTCA  aggactcgcgGATTGTATACAACCTGTCGAGCCCG  gtatacaatcCGCGAGTCCTCTTGATGC  ttgcatgcctgcaggtcgacTATCATAGATCTCGACAATGTCTTCAATC  ATGGCGAAGAACACCAAC  TCCACCGAGTCATGAAGG  taacagggtaatctgaattcCGTTTGTTTTGAAGAGATC  cgaccgacgaCAGTTGAAAGTTTCTTGAC  ctttcaactgTCGTCGGTCGGTAAAACAGAAAAC  ttgcatgcctgcaggtcgacTTTTGCCGGCCTGCTGGT  AAACGGGATGGCACAAGC  GAGCCGAAGTTCTTCATC  taacagggtaatctgaattcGCCGACGGCGGTACCCGC  gcgattgccgGGCGCGACCCTCTCGCGC  gggtcgcgccCGGCAATCGCTGCGTTGC  ttgcatgcctgcaggtcgacGGTGGCGAAGAAGGCTTC  AGTGACCTGGATGTCTTG  TCACCGCCACAGTCTTTC  taacagggtaatctgaattcAAAGAGGCCAGCACGAAG  agcgcttcgcGACTTCAATCTCTGACTGATTGG  gattgaagtcGCGAAGCGCTCCTGCTTC  ttgcatgcctgcaggtcgacGAAATCCGCGGCACATTGC  CGTAACCCAGTGCAATCG  ATGCGCCAACTGGAAGAG  ttgcatgcctgcaggtcgacAGTCAAAAACAAAGTTTGAC  taacaacgccATACACATCACAAAGCCTG  tgatgtgtatGGCGTTGTTAAGCTCCTTTTAAAC  taacagggtaatctgaattcCAATGTTGACGTGCTGCC  GCGCCATAATCAATGCTG  TACACATCCCTGCTCAAC  AGCGTTTGCTCGAAGAAGTG  GCAATAGATCCGGTAGGG  taacagggtaatctgaattcATGCAAGGGCTTTGGGTTC  cggtggtctgTGGACTCTCCGTGTGACC  ggagagtccaCAGACCACCGGGGCCGCA  ttgcatgcctgcaggtcgacCAAGGCGGCTGACATTTTTCACTCCC  CCTTGAATCGGTGTTGAG  GTCCAGGCCTAAGATCTC  taacagggtaatctgaattcTGGCGCCCATGTCGCTGC  gggagcatccGGCCGCTGTAAACCCGTCG  tacagcggccGGATGCTCCCTGCTGATATC  ttgcatgcctgcaggtcgacTTCTATCACCGCCTCAGC  GTACTGCGACTGGTACTC  CTGTTCCTCGACGAAGTG |

Supplementary Table 2. Correlation of OD to CDW values for all applied surfactant producer strains

| **Strain** | **Biomass conc. [g_CDW_/L] corresponding to OD_600_ = 1** |
| --- | --- |
| *P. putida* KT2440 SK4 | 0.343 |
| *P. putida* KT2440 ∆*lapF*_RL | 0.348 |
| *P. putida* KT2440 ∆*lapA*∆*lapF*_RL | 0.324 |
| *P. putida* KT2440 ∆flag_RL | 0.305 |
| *P. putida* KT2440 GR20_RL | 0.343 |
| *P. putida* KT2440 KS3 | 0.315 |
| *P. putida* KT2440 ∆*lapF*_HAA | 0.316 |
